# Surgical and conservative treatment of patients with congenital scoliosis: α search for long-term results

**DOI:** 10.1186/1748-7161-6-12

**Published:** 2011-06-04

**Authors:** Angelos Kaspiris, Theodoros B Grivas, Hans-Rudolf Weiss, Deborah Turnbull

**Affiliations:** 1 Department of Trauma and Orthopaedics,"Thriasio" General Hospital - NHS, G. Gennimata av, Magoula 19600, Attica, Greece; 2 Department of Trauma and Orthopedics, Scoliosis Clinic,"Tzanio" General Hospital - NHS, Tzani and Afendouli 1 st,Piraeus 18536, Greece; 3 Orthopedic Rehabilitation Services Alzeyer Str. 23, D - 55457 Gensingen, Germany; 4 Clayponds Hospital, Ealing PCT, Sterling Place W134RN, London, UK

## Abstract

**Background:**

In view of the limited data available on the conservative treatment of patients with congenital scoliosis (CS), early surgery is suggested in mild cases with formation failures. Patients with segmentation failures will not benefit from conservative treatment. The purpose of this review is to identify the mid- or long-term results of spinal fusion surgery in patients with congenital scoliosis.

**Methods:**

Retrospective and prospective studies were included, reporting on the outcome of surgery in patients with congenital scoliosis. Studies concerning a small numbers of cases treated conservatively were included too. We analyzed mid-term (5 to 7 years) and long-term results (7 years or more), both as regards the maintenance of the correction of scoliosis and the safety of instrumentation, the early and late complications of surgery and their effect on quality of life.

**Results:**

A small number of studies of surgically treated patients were found, contained follow-up periods of 4-6 years that in the most cases, skeletal maturity was not yet reached, and few with follow-up of 36-44 years. The results of bracing in children with congenital scoliosis, mainly in cases with failure of formation, were also studied.

**Discussion:**

Spinal surgery in patients with congenital scoliosis is regarded in short as a safe procedure and should be performed. On the other hand, early and late complications are also described, concerning not only intraoperative and immediate postoperative problems, but also the safety and efficacy of the spinal instrumentation and the possibility of developing neurological disorders and the long-term effect these may have on both lung function and the quality of life of children.

**Conclusions:**

Few cases indicate the long-term results of surgical techniques, in the natural progression of scoliosis. Similarly, few cases have been reported on the influence of conservative treatment.

In conclusion, patients with segmentation failures should be treated surgically early, according to the rate of deformity formation and certainly before the pubertal growth spurt to try to avoid cor- pulmonale, even though there is lack of evidence for that in the long-term. Furthermore, in patients with formation failures, further investigation is needed to document where a conservative approach would be necessary.

## Background

Noted in about 1 in 1000 births, congenital scoliosis is the most common congenital spinal disorder, followed by congenital kyphosis and lordosis [1 - 2]. The vertebral disorders that cause Congenital Scoliosis may be due to either failure of formation or failure of segmentation or a combination of these, leading to a mixed deformity [1 - 4]. Complete failure of formation leads to hemivertebrae with the absence of one pedicle and a region of the vertebral body, while incomplete failure of formation leads to a wedged vertebra [1 - 4]. Both types of malformations may be lateral, causing scoliosis; posterolateral, causing lordoscoliosis; dorsal, causing lordosis; anterolateral, causing kyphoscoliosis or ventral, causing kyphosis [1]. When the anterior part of the vertebra is deficient, while the dorsal part is not malformed, kyphoscoliosis, especially in the lumbar spine, is common.

For congenital scoliosis, the most severely progressive deformities are those due to unilateral defects of segmentation [4].

Congenital Scoliosis is believed to be associated with any damage caused to the foetus during its intrauterine development and the formation of the spine, between the 5th and 8th week of gestation. Thus, it is often associated with other disorders, such as congenital heart disease, spinal cord dysraphism and congenital kidney disorders [3,4]. Approximately 10-15% of patients with congenital scoliosis present congenital heart problems, such as ventricular septal defects, tetralogy of Fallot or transposition of great vessels [5,6]. In addition, the severe restriction of pulmonary function in cases of large curves raises the suspicion of the coexistence of hypoplastic lung development [6,7].

According to Winter, congenital deformities can be very benign to incredibly severe, can result in death from cor pulmonale, cause paraplegia, and can be associated with multiple other problems. They are challenging to paediatricians, physicians, and surgeons because of the high frequency of associated medical problems, the existence of large curves at a young age, and the relative rigidity of the curves compared with those of idiopathic and neuromuscular patients [4].

As Batra and Ahuja [8] report, congenital scoliosis remains an interesting and challenging diagnostic problem. Vertebral absence, partial formation, or lack of segmentation may cause asymmetrical growth and resultant deformity.

The high frequency of associated anomalies within and outside the spine necessitates a detailed history and physical examination. Maternal, perinatal history, family history, and developmental milestones must be fully explored. The physical examination of both the skin in the spinal midline to investigate the existence of nevi, haemangiomas or hairy patches, is deemed necessary, as they are sings of underlying spinal dysraphism. In addition, spinal examination should focus on the cervical pars, due to the connection of congenital scoliosis with the Klippel-Fail syndrome. Moreover, a neurological examination is necessary to examine the existence of latent ataxia or myelopathy [1]. This often coexists with other syndromes such as Alagille, Jarco Levin, Joubert, basal cell naevus and diabetic embryopathy. It may be associated with musculoskeletal disorders such as Sprengel's deformity, clubfeet, or DDH [6].

Plain radiographs remain the standard for the diagnosis of congenital anomalies and measuring curve magnitude, progression and perhaps the growth potential of the vertebral anomaly. Preoperative CT scans define the anatomy and avoid any unexpected intraoperative posterior element deficiencies. MRIs can exclude associated conditions of the spine, cranio-cervical junction, and viscera.

Statistically, 25% of curves do not present progression, 25% present mild progression while 50% present rapid deterioration and require treatment [2-11].

Knowledge of the natural history of congenital scoliosis is important because it can determine its management approach. McMaster and Ohtsuka [12] were the first to focus on the natural progression of congenital scoliosis and define the risk of further deterioration (progression) in detail, in relation to four key factors: the type of congenital anomaly, its location on the spine, the patient's age at the onset of the disorder and solitary or multiple curves. These disorders are divided primarily into four main categories and concern failure of formation, failure of segmentation, mixed defects and complex unclassifiable defects [12,13].

In the first category of failure of formation, this could occur as wedge vertebra, located in the lower thoracic and thoracolumbar regions with a relatively low rate of progression of 1° to 2° per year. In addition, this category includes simple unsegmented hemivertebra, which do not have a potential for growth and subsequently a minimal risk of progression. Usually, curves are less than 30° at maturity. In the case of semi segmented / fully segmented and multiple hemivertebrae, the risk of progression depends on their location, number, and degree of segmentation. Specifically, the upper thoracic hemivertebra progress on average 1° to 2° per year before the age of 10 years and 2° to 2.5° after this age. However, when detected in the lower thoracic region, they show a more rapid progression of 2° per year before puberty and 2.5° to 3° after this. In the thoracolumbar area, the rate of progression is much faster, from 2° to 2.5° per year before puberty, to 3.5° per year after that, resulting in substantial trunk imbalance. By contrast, lumbar hemivertebrae present a lower degree of progression compared to thoracic [12,13].

The second category concerns failure of segmentation. Block vertebrae are usually multiple with a small potential for growth and a slow rate of progression (less than 1° per year). The extent of the unilateral unsegmented bar and its position determine its natural development. In the upper thoracic spine, the rate of progression is 2° per year before puberty and 4° after. In the lower thoracic area, it is 5° and 6.5° respectively. In the thoracolumbar area, the rate of progression increases to 6° and 9° respectively, while in the lumbar area it is about 5° per year [12,13].

The third category concerns mixed defects, which may involve a unilateral unsegmented bar and a contralateral hemivertebra. These types of disorders occur more frequently in the thoracic spine and are the most severe of all scoliosis disorders. They present rapid deterioration of up to 14°per year and result in clinical trunk shortening, limb length discrepancy and severe cosmetic deformity [12,13].

The fourth category concerns complex mixed pattern anomalies that do not belong to the above categories and whose development it is particularly difficult to predict. Overall, however, thoracolumbar apex curves exhibit a greater tendency of deterioration in relation to thoracic or lumbar [12,13].

The recognition of curves with a poor prognosis at an early stage is essential to prevent curve progression and possible neurological complications. The aim of surgery is to achieve a straight spine and a physiological sagittal profile while maintaining flexibility, to inhibit progression of the curve with a short fusion segment preserving normal spinal growth as much as possible. Developments in gene research continue to be promising and may potentially lead to early detection of congenital vertebral malformations.

The therapeutic options in cases of congenital scoliosis include conservative or surgical approaches. Of course, few data exist on conservative management, although it seems that patients with specific types of segmentation failures, like unilateral unsegmented bars, will not benefit from conservative treatment, while the same applies to formation failures with curves of >20 degrees in infancy [14]. Of course, cases with formation defects such as non-incarcerated, semicancerated or incancerated hemivertebrae receive a variety of treatments ranging from observation to brace treatment or surgical intervention. In general, most congenital scoliotic curves are not flexible and therefore are resistant to repair with bracing. For this reason, the use of braces mainly aims to prevent the progression of secondary curves that develop above and below the congenital curve, causing imbalance. In these cases, they may be applied until skeletal maturity [6].

In addition, early surgery is suggested even in mild cases with formation failures in the first three years of life, although there are reports that, in this group of patients, a conservative approach might be beneficial [14,15].

According to Repko et al, congenital scoliosis due to failure of formation or segmentation is indicated for surgical treatment at a young age. Its early detection and the subsequent surgical correction of the curve leads to long-term maintenance of a compensated spine. Instrumented hemivertebra excision provides the highest rate of correction, particularly if carried out before the age of 3 [16].

Other authors believe that congenital scoliosis correction surgery should be performed early, before the development of severe local deformities and secondary structural changes, especially in patients with expected deterioration [17,19].

Generally, the choice of surgical approach depends on the type of anomaly, the degree of deformation and the age of the patient [20].

**Figure 1:**
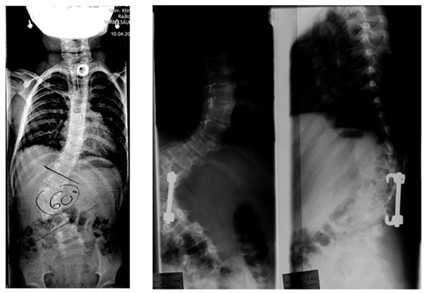
**Progression after early operation for severe hemivertebra at the age of 6 years**. After surgical intervention at the age of 6 years, there was a significant progression of scoliosis (60 to 90 degrees) and kyphosis as well at the last follow-up at the age of 11 years, the patient still being premenarchial.

The most common surgical techniques used are in situ fusion, convex hemiepiphysiodesis, and hemivertebra excision. In situ fusion is indicated as the most reliable and safe operation for congenital spinal disorders. Ideal candidates for this are patients with a fully segmented hemivertebra, with no associated deformity. The classic indication is a patient with a unilateral bar or a unilateral bar with contralateral hemivertebrae, diagnosed early, before any significant deformity [20-23]. In contrast, in convex hemiepiphysiodesis, the classic indication is a patient with segmented hemivertebra without any associated deformity. It is not considered reliable in patients requiring correction and is contraindicated if there is no concave growth potential [20,21]. In addition, hemivertebral excision remains an attractive surgical option in cases where the hemivertebra causes progressive curve and deformity (Figure 1**)**. It is considered ideal for children under the age of five, with fully segmented hemivertebrae in the thoracolumbar junction, lumbar spine, or lumbar sacral spine. The hemivertebra can cause problems when located in the cervico-thoracic or cervical region from which it could be removed [20,24]. Hemivertebrae may be resected by an anterior-posterior or by a posterior procedure only [20,21].

Many authors regard the surgical procedures performed as safe in the short-term [20-26]. The procedures described as being safe were mainly transpedicular hemiepiphysiodesis [26] and excision of the hemivertebrae [27].

Another operation described as very safe is expansion thoracostomy and insertion of a vertical expandable prosthetic titanium rib (VEPTR) [28-31]. In addition, according to Hell et al, VEPTR is presently considered superior to any other method for the treatment of small children with progressive scoliosis in cases where deformities with rib fusions are combined with a constricted thorax with chest expansion and poor development of the pulmonary parenchyma, leading to thoracic insufficiency syndrome [29].

On the other hand, many long-term disadvantages of early surgery have been reported.

**Figure 2:**
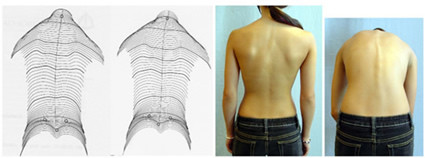
**No progression in a patient with 26° thoracic and 21° lumbar and failure of formation from the age of 10 (premenarchial) to the age of 14 (2 years postmenarchial)**. No cosmetic difference at the age of 10 (left Formetric^® ^surface scan), at the age of 12 (right Formetric^® ^surface scan) and at the age of 14 years at Risser 3-4 (clinical pictures on the right), when treatment and observation stopped. Although the clinical pictures and scans cannot be compared well, when looking at the outline of the figures no change in lateral deviation is visible.

**Figure 3:**
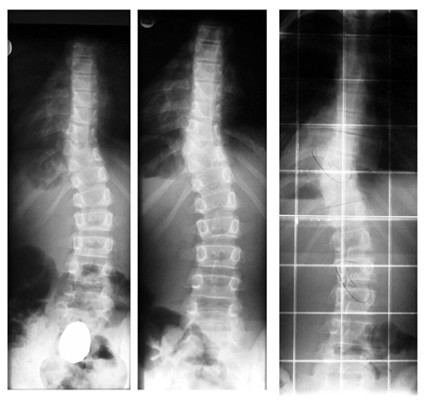
**No progression in a patient with nearly 26° thoracic and 21° lumbar curve and failure of formation from the age of 10 (premenarchial) to the age of 14 (2 years postmenarchial)**. At 10 years, a girl with congenital scoliosis appeared with a 26° thoracic and 20° lumbar curve as can be seen on the left (the same as she had at the age of 8). At 12 years (middle) she had 22° and 25° curve and at the end of treatment at the age of 14 (right) she had 22° thoracic and 20° lumbar curve, respectively. She has been treated conservatively; however even without treatment, she would have overcome the pubertal growth spurt without significant progression.

Νatural history of congenital scoliosis may in part be undesirable, but not all patients with congenital scoliosis should be regarded as progressive [4] and some will respond to conservative management (Figures 2,3) [12]. Scientifically, we should demonstrate that intervention (surgery) alters the natural history of congenital scoliosis in a favourable and reproducible manner. We should also demonstrate that the long-term side effects of spinal fusion in patients with congenital scoliosis are not detrimental, so that the risk-benefit ratio favours the intervention over the condition's natural history, while it has already been demonstrated that the rate of complications may be relatively high in the long-term [32-38].

The purpose of this systematic review of PubMed literature was to identify the mid- or long-term results of spinal fusion surgery and conservative treatment in patients with congenital scoliosis.

## Methods

The medical literature was searched exhaustively to access all articles (mainly in English) pertaining to the mid- or long-term results of the conservative or surgical treatment of congenital scoliosis.

The subject search used a combination of controlled vocabulary, MeSH headings and free text terms based on the following search strategy for searching MEDLINE:

Congenital scoliosis; spine surgery; scoliosis surgery; spondylodesis; spinal instrumentation and spine fusion; bracing; conservative treatment; long-term results or long-term follow-up.

The electronic databases searched were:

1. MEDLINE (1966 - present)

2.EMBASE (1980 - present)

3.CINAHL (1960 - present)

4.AMI

5.AMED (1985 - present)

In addition, the reference lists of all eligible trials, key textbooks and previous reviews were searched for additional studies.

Specific exclusion and inclusion criteria were followed for the selection of studies. The examined studies included all types of retrospective and prospective studies, obviously reporting on the outcome of scoliosis surgery in patients with congenital scoliosis.

The examined population included patients with congenital scoliosis of various aetiologies, subjected to surgical or conservative intervention. Specifically, studies with a mean follow-up period of 5 to 7 years were considered to concern mid-term results, while those with a follow-up period of 7 years or more were considered to concern long-term results.

Written informed consent was obtained from the patients for publication of this report and accompanying images. A copy of the written consent is available for review by the Editor - in -Chief of this journal.

## Results

Despite the large number of studies concerning both the pathogenesis, and the surgical and conservative treatment of congenital scoliosis, very few focused on the long-term results of therapy.

Ten (10) studies (Table 1) met the inclusion criteria we had set for follow-up in cases of surgical treatment of congenital scoliosis. Two concerned the mid-term results and the remaining eight the long-term results [10,37,39-44], and in most of the cases presented, skeletal maturity was not yet reached [10,41-44]. In addition, few studies reported cases followed retrospectively over 36-51 years [37, 39, 40, and 45]. Furthermore, we studied the quality of life in patients who had undergone surgery on a long-term basis [46]. Finally, our study included newer experimental data on the effects of surgery in animal models [47-50]. The general search on "congenital scoliosis" however produced some other studies containing follow-up data with follow-up times between 3 and 4.5 years [17,51-54] but only one with a patient cohort beyond skeletal maturity [55]. Studies with a follow-up of less than 5 years were not included in this review.

**Table 1:** Follow - up of surgically treated patients with Congenital Scoliosis

Study	Sample	Intervention	Follow - up	Outcomes	Results
Elsebai 2011	19 children (7 males, 12 females) Mean age: 6.9 years 5 failure of segmentation 4 failure of formation 5 mixed 5 unclassified	VEPTR	5 years (2 to 7 years)	Preoperative mean Cobb angle:66° (range: 40 - 95°) Postoperative mean Cobb angle: 45° (range: 18 - 78^0 ^)	No neurological complications 14 complications at 8 pts 5 rod breakage 3 proximal construct dislodgment 2 implant failures 1 painful protruding anchor 2 pulmonary complications 1 postop deep infection

Takayama 2009	8 children (3 males, 5 females) Mean age:13.7 years	Posterior fusion Harrington instrumentation:7 Dweyer: 1	23.1 years	Preoperative mean Cobb angle:54.5° Postoperative mean Cobb angle: 30.5° Cobb angle at the recent follow up: 36.8° Loss of correction: 6.3°	SF - 36 Questionnaire score Physical function: 49.4 Role physical: 51.1 Bodily pain: 47.8 General health: 49.1 Vitality: 47.6 Social function: 57.1 Role-emotional: 55.0 Mental health: 51.8 SRS-22 Total score Function/activity: 4.20 Pain:4.45 Self-image appearance:3.48 Mental health: 3.93 Satisfaction with management: 3.75

Ruf 2009	41 children 19 males, 22 females Mean age: 3.5 years Range: 1.5 - 7 years Children without bar formation 28 pts, 31 hemivertebrae T1 - T9: 7 pts T10 - L2: 18 pts L3 - L4: 6 pts All were non incarcerated, 19 fully segmented, 12 semisegmented Children with bar formation 13 pts, 20 hemivertebrae 5 pts with double hemivertebrae 1 pt with 3 hemivertebrae on the same side, 10 pts with controlateral rib synostosis, Thoracic spine: 10 pts, Thoracolumbar spine: 2 pts, Lumbar spine: 1 pt	Hemivertebrae resection by a posterior approach and transpedicular instumentation	6.5 years	Children without bar formation Preoperative mean Cobb angle: 36.1° Postoperative mean Cobb angle: 7.1° Cobb angle at the recent follow - up: 6.8° Children with bar formation Preoperative mean Cobb angle: 69.2° Postoperative mean Cobb angle: 23.3° Cobb angle at the recent follow - up: 20.8°	Children without bar formation No neurologic complications Implant failure in 3 cases (revision performed), Children with bar formation No neurologic complications Revisions performed in 3 pts due to haematomas, deep infection, development of new deformities, In one pt the rods were removed because of increased lordosis

Winter 2009	7 children 4 males, 3 females Mean age: 7 years Range: 1 - 17 years All the patients had segmentation defects Thoracic spine: 3 pts, Thoracolumbar spine: 2 pts, Lumbar spine: 2 pts	5 treated with posterior spine fusion, 2 left untreated	50 years	Preoperative mean Cobb angle: 66.5° Postoperative mean Cobb angle: 41.8° Cobb angle at the recent follow - up: 49.3°	Neck pain in 1 pt, Signs of cor - pulmonale in 1 pt, Low back pain and shoulder pain in 1 pt,

Chen 2009	21 children, 8 males, 13 females Mean age: 20.1 years Range: 12 -42 years 20 pts with single level hemivertebrae, 1 pt with hemivertebrae and controlateral unsegmental bar, Thoracic spine: 7 pts, Lumbar spine: 14 pts	19 treated with posterior instrumentation fusion, 2 combined with anterior hemivertebrae excision	9 years Range: 3 - 18 years	Preoperative mean Cobb angle: 45.28° Postoperative mean Cobb angle: 32.57° Cobb angle at the recent follow - up: 36°	No neurological complications, Low back pain in 3 pts, Not successful curve correction in 3 pts, Loss of reduction in 1 pt, Broken screws in 1 pt

Vitale 2008	21 children 9 males, 12 females Mean age: 4.9 years, Range: 1 - 10 years Hemivertebrae: 11 pts, Unsegmented bar: 1 pt Complex: 9 pts Above the thoracolumbar junction: 12 pts, Below the thoracolumbar junction: 9 pts	3 treated with posterior in situ fusion, 3 treated with posterior spinal fusion+ instrumentation, 7 treated with PSF+ instrumentation, 7 treated with in situ fusion anterior and posterior, 1 treated with anterior fusion + instrumentation	7 years Range: 3 - 13 years	Preoperative mean Cobb angle: 53.6° Cobb angle at the recent follow - up: In pts with thoracic fusion: 41.6° In pts with not thoracic fusion: 4.4°	Pulmonary Function Tests(PFT) FVC: 74.4% FEV: 73% LTC: 88.5% VC: 75.6% Child Health Questionnaire(PF-28) Physical functioning: 78.3 Social behavioral role: 88.8 Social-physical role: 90.4 Bodily pain/discomfort:71.4 Global behavior: 74.4 Mental health: 76.9 Self-esteem: 82.1 General health perceptions: 59.3 Physical summary: 44.5 Psychosocial summary: 50.6 Parental impact - emotional:68.4, Parental impact time: 85.7, Family activities: 84.5 Family cohesion: 67.6

Bollini 2006	34 children 16 males, 18 females Mean age: 3.5 Range: 1 - 9.8 years Hemivertebrae in all pts Fully segmented: 12 Semi segmented: 22 Thoracic spine: 3 pts Thoracolumbar spine: 21 pts Lumbar spine: 10 pts	Hemivertebrae resection by double approach	7.1 years Range: 2 - 14.6 years	Preoperative mean Cobb angle: 40.4° Postoperative mean Cobb angle: 24.6° Cobb angle at the recent follow - up: 26.9°	Postoperative complications Paraparesis in1 pt/reoperation, Wound infection in 2 pts, Respiratory infections in2 pts, Pneumothorax in 1 pt, Pleural effusion in 1 pt Late complications Abdominal wall hernia in 1 pt, Pseudarthrosis in 5 pts, Breakage of the Harrington rod in 1 pt, Grafting revision in 2 pts, Sepsis / implant removal in 1 pt, Progression of the curve in 6 pts, Progressive kyphosis in 1 pt (treated with vertebral osteotomy and arthrodesis), Implant removal in 11 cases

Winter 2004	1 child (case report) Male 12 months Unilateral unsegmented bar with nonsegmentation of the ribs at the same side, Thoracic Spine(T4 - T7)	Posterior spine fusion	44 years	Preoperative mean Cobb angle: 37° Postoperative mean Cobb angle: 28° Cobb angle at the recent follow - up: 32°	VC: 70%, Neck pain with slightly weak left deltoid, Low back pain, Multilevel degenerative changes in the lumbar spine

Marks 1995	53 children 27 males, 26 females Mean age: 6.5 years Range: 2 - 12 years 30 pts with fully segmented non-incarcerated hemivertebrae, 4 pts with unsegmented bars, 7 pts with unsegmented bars with hemivertebrae, 12 pts with complex types Thoracic spine: 33, Thoracolumbar spine: 11, Lumbar spine: 10 Lumbo-sacral: 1	Anterior and posterior convex epiphysiodesis	9 years Range: 3 - 22.5 years	Unilateral unsegmented bars Preoperative mean Cobb angle: 47.5° Cobb angle at the recent follow - up: 74.5° Unilateral unsegmented bars and hemivertebrae Preoperative mean Cobb angle: 49° Cobb angle at the recent follow - up: 52° Hemivertebrae Preoperative mean Cobb angle: 41° Cobb angle at the recent follow - up: 35° Complex anomalies Preoperative mean Cobb angle: 74° Cobb angle at the recent follow - up: 90°	Neuroapraxias in 3 pts (1 of an intercostals neure and 2 of the lateral cutaneous neure of the thigh)

Studies highlighting the complications and mid - term and long term outcomes of surgical interventions in various types of congenital scoliosis

## Discussion

Spinal deformity in early childhood has a poor prognosis, as progression and severe respiratory compromise are probable. Treatment is difficult, as patients even with idiopathic scoliosis frequently do not respond to bracing, and surgery is sometimes performed in childhood in an attempt to control relentless progression. This entails the risk of continued deformation during subsequent growth, and the surgical procedures have been adapted in an attempt to minimize this. Children undergoing spinal fusion for progressive and severe deformity have undergone sequential topographic scans, which show that, despite measures to control the rib hump, progression after surgery is fairly common [56]. In agreement with two recent reviews [57,58], this study shows that spinal fusion surgery cannot improve all symptoms of scoliosis in the long-term. Additionally, the risks of such surgery are commonly underestimated [38,59].

A first question concerns the safety and efficacy of spinal instrumentation in children with congenital spinal anomalies. This is a subject of general debate and many believe that congenital curves should not be instrumented while others disagree [21,60]. The purpose of this technique is not so much to fully align the spine, but to achieve safe correction and balance for the patient [21]. Although there are reports of complete safety in use in childhood or infancy [61], especially using new-generation implants [62], some studies have identified potential risks. In their sample, Ayvaz et al. [35] report an overall complication rate of 31%, including neurological compromise in 2 patients (9%) of which one developed paraparesis. The authors concluded that spinal instrumentation was effective in the control of deformities, but with a relatively high rate of complications. A recent study by Qiu et al also shows that 11 (2.89%) out of 381 patients with congenital scoliosis who had undergone surgery developed neurological disorders, ranging from amytrophy, lower limb radicular pain, sphincter dysfunction to paraparesis of both lower limbs [63]. However, as regards high complication rates, the ideal solution for managing the congenital cases is still to prevent the progression of the curve through early intervention using the optimal surgical approach for each patient.

On the other hand, the current expandable spinal implant systems appear effective in controlling progressive early onset scoliosis (EOS), allowing for spinal growth, and improving lung development. All however have a moderate complication rate, especially rod breakage and hook displacement [36].

While early spinal fusion may halt progressive deformity in young children with scoliosis, it does not seem to facilitate lung growth and, in certain children, can result in thoracic insufficiency syndrome. This is because the growth of the spine is very high before the age of 5 years, and spinal fusion in this early period of spinal growth has significant effects on respiratory physiology [64 - 65]. Patients with proximal thoracic deformity who require fusion of more than four segments, especially those with rib anomalies, are at the highest risk of developing restrictive pulmonary disease [32]. Furthermore, patients with congenital scoliosis subjected to early spinal fusion have significantly reduced lung function. According to Vitale et al (Table 1)[33], the average FVC (Forced Vital Capacity), FEV (Forced Expiratory Volume), TLC (Total Lung Capacity) and VC (Vital Capacity) of these patients are significantly reduced compared with healthy children, with scores of 74, 73, 89 and 76 respectively. Indeed, if we focus on the children who have undergone thoracic fusion, these values are even lower (64, 64, 81 and 67 respectively). Furthermore, the patients with thoracic fusion had significantly lower physical functioning and physical summary compared with healthy children. Patients with lumbar fusion scored significantly lower in parent impact - emotional domain compared with healthy children. Both patients with thoracic and non-thoracic fusion tended to have significantly lower scores in general health perceptions [33]. Radiological measurements too showed that lung growth was reduced in cases of early posterior spinal fusion, without however a great difference in lung function compared with untreated patients. However, this happens in the middle of follow-up and more accurate information could be gained through measurements taken at the end of growth, where the long-term effects would become more precise [66].

Apart from clinical functionally indicators, quality of life is also affected. Compared with healthy peers, congenital scoliosis patients treated with early spinal fusion present differences in the PFT and Quality of Life (QOL) scores at 6.9 years follow-up. Patients with thoracic fusions had shorter spines, worse pulmonary function, and more pain than the non-thoracic fused. In addition, they presented lower physical functioning and physical summary values, compared with healthy children [33]. A contrary view is expressed with the use of the SRS-22 and SF-36 questionnaires, in which the total scores at follow-up over more than 16 years showed no significant differences compared with normal controls. In addition, there are no differences in working, marital and labour conditions, except for a more frequent need for caesarean sections during pregnancy in those who operated for congenital scoliosis, compared with the overall population or idiopathic scoliosis [46]. The results may support alternatives to early spinal fusion, such as growing rods, epiphysiodesis, and distraction thoracoplasty [33], and, possibly, a conservative approach [14].

Crankshafting was observed in 15% of the patients, more often with larger curves and earlier fusions [34].

Thompson et al (Table 1) [41], reviewed thirty patients after surgical intervention of which 16 male and 14 female. Follow-up was at minimum 3 years (average 8 years 10 months; range, 3-22.5 years). Nineteen patients were skeletally mature, and the mean age of the remaining 11 was 11.75 years. A reversal of the Cobb angle was noted in 23 patients; in five, it was delayed while two patients presented progression. The results related to the age at which the surgery was conducted and the position of the hemivertebrae, with much better results when they were located in the lumbar region.

Marks et al (Table 1)[42], examined 53 patients (27 male, 26 female) with a minimum follow-up period from surgery of 3 years (mean 8.8 years, range 3-22.5 years). The types of vertebral anomalies encountered were 4 unsegmented bars, 7 unsegmented bars with hemivertebrae, 30 hemivertebrae - of which 2 double hemivertebrae - and 12 complex-unclassifiable patterns. Of these, 34 were skeletally mature when reviewed. Clinical assessment and sequential measurement of Cobb angles were used to chart the course of the deformity following convex epiphysiodesis surgery. In cases with unsegmented bars, there was no significant improvement of more complex anomalies; initially there was a small degree of postoperative reduction in the rate of the deformity's progression, while ultimately the Cobb angle increased from 61° to 70°. In contrast, the rate of progression reversed or slowed in 97% of hemivertebra patients following surgery, producing a change in mean Cobb angle from 41° preoperatively to 35° post-operatively. In this study, the younger age of surgery and the position of the hemivertebrae in the lumbar spine were associated with better results.

Winter and Smiths study a case with a 44-year follow-up after surgery at the age of 1 year for congenital scoliosis (Table 1**)**. Low back pain began 22 years after surgery (at 23 years) and cervical pain 24 years after surgery (at 25 years) [37]. Anterior cervical discectomy and fusion plus posterior fusion of two disc levels were necessary at the age of 36 years. Continued low back pain resulting from multilevel degeneration caused major disability. It was never ascertained whether the problems were due to the surgical intervention or should be regarded as part of the natural history of this individual.

Te Chen and Wang studied single level hemivertebrae in 22 patients, 8 male and 14 female, over an average of 19.3 years (Table 1**)**. The levels of the hemivertebrae were from T8 to L5. Spine-related anomalies were noted in five cases, including one case of skin dimple, one case of cervical rib and three cases of spinal bifida. Other congenital anomalies were noted in 3 cases with congenital heart diseases, 2 cases with genitourinary anomalies, 2 cases with gastrointestinal anomalies, one case with craniofacial deformity and 2 cases with mental retardation. Preoperatively, the 19 patients who underwent posterior instrumentation surgery, showed curves between 24° and 65°, which improved postoperatively at a range between 17° and 52°. No neurological complications were observed after surgery. The complications that occurred were one case with superficial wound inflammation, 1 case of loss of repair and one case with broken screws. Nevertheless, during the follow-up, there was a progression from 20° to 53°. Three skeletally mature patients experienced severe back pain, which improved after fusion in situ [67]. Interestingly, in a 1.5 year-old girl with hemivertebrae at the level of T3 and a 21.5° curve, which followed conservative treatment, marked only a 5° curve progression in the 6-year follow-up [67].

Another study with long-term follow-up of thoracolumbar hemivertebrae resection by double approach, by Bollini et al. [51] involved 21 patients with an average follow-up of 8.6 years and an average age of 11.8 years (Table 1**)**. Of these, four were skeletally mature, with Risser signs of 4 and 5, while the rest immature with Risser signs of 0 to 3. The pre-operative curve was 32.9°, the operation it was 11.2° and, in the last follow-up 9.4°. Immediate complications included mild radiculopathy, followed by a complete recovery and superficial wound infections. In one patient, the spinal implants were removed due to rod fracture, while one patient presented hook displacement, which necessitated hook reinsertion. In addition, a patient presented pseudarthrosis that required bone grafting and revision of posterior instrumentation to treat progressive kyphosis.

The last study, by Winter and Lonstein (Table 1**)**, with a mean follow-up of 51 years involved seven case reports on congenital scoliosis [45], some treated surgically and others left untreated. Specifically, individuals who had undergone surgical treatment with posterior spinal fusion showed stabilization of curves, with a vital capacity of around 70%, but with major complaints concerning the neck, lower back, and shoulders. In particular, five patients (3 boys and 2 girls) in this study, suffering from congenital scoliosis with unilateral unsegmented bars, were operated (spinal fusion). The age at diagnosis was 3 to 6 years and that of follow-up 44 to 59 years. The curves were located in the chest and the thoracolumbar region, ranging from 37° to 115°. Of those who did not follow any treatment [a 13 year-old girl with 40° congenital thoracolumbar scoliosis and a 17 year-old boy with 60° (T1/5) and 80° (T5/12) double thoracic scoliosis] the first was severely decompensated to one side while the second did not show any significant curve progression.

Another retrospective study, by Ruf, Jensen et al, relating to hemivertebra resection, examined the impact of intervention in 41 patients who underwent 51 consecutive hemivertebrae resections (Table 1). Resections were performed by a posterior only approach with transpedicular instrumentation. Patients were 22 girls and 19 boys divided into 2 groups according to the prognosis of the disease. The first group comprised 28 patients with 31 hemivertebrae without bar formation, in the thoracic region (T1-T9) in 7 cases, in the thoracolumbar region (T10-L2) in 18 cases and in the lumbar region (L3-L4) in 6 cases. All hemivertebrae were non-incarcerated, 19 were fully segmented and 12 hemisegmented. The second group consisted of 13 patients with 20 hemivertebrae. Five of these had double hemivertebrae and 1 patient had 3 hemivertebrae on the same side. 10 cases were located in the thoracic spine, 2 in the thoracolumbar region and 1 case in the lumbar spine. The contralateral bar comprised up to seven segments. Ten patients showed contralateral rib synostosis. The average period of follow-up was 6 years with a range of approximately 10 months to 16 years. None of the two groups presented any neurological disorders. In the first group, the average curve was 36.1° before surgery, 7.1° after and 6.8° at the last follow-up. In 3 cases, the implant failed and needed revision, while in 3 patients a convex pedicle was overloaded and broke. In the second group, the average curve was 69.2° before surgery, 23.3° after and 20.8° at the last follow-up. Three cases needed revision due to hematoma, infection, and development of new deformities. Of course, a key issue discussed by the authors concerns the growth deficit after spinal fusion in these young children [68]. Certainly according to DiMeglio [69] we may expect a growth deficit at the end of growth after fusion of 5 vertebrae, when surgery is performed at the age of 2 years.

In the papers cited in this review, spinal surgery in patients with congenital scoliosis is regarded as a safe procedure [19, 25 - 31, 51, 55] and many authors claim that surgery should be performed as early as possible to prevent the development of severe local deformities and secondary structural deformities that would require more extensive fusion later [17 -19, 51, 55]. On the other hand, severe late complications have also been described in literature [32-35].

In addition, newer experimental data give further momentum to the use of surgical techniques, specifically regarding the possible consequences of anterior spinal fusion in the development of the vertebral canal, as the ring apophyses are not the only growth centres of the vertebral body. The neurocentral cartilage located in the posterior two thirds of vertebrae is responsible for the development of pedicles and posterior vertebral bodies. Compressive forces along this may lead to iatrogenic vertebral stenosis. This was demonstrated in immature porcine experimental models studying the effect of anterior spinal fusion in the development of the spinal canal. Based on the Heuter-Volkman principle, these forces may inhibit the growth of the vertebral canal at the fused levels [47-50]. Moreover, this led to the development of kyphosis due to decreased growth of the anterior column and continued growth of the posterior column. However, the authors stress that spine surgeons should take into account that the direct identification of these findings in clinical practice is difficult [47].

Recently, Hefti [57] demonstrated that hemivertebra resection bears significant risks, while the VEPTR procedure appears relatively safe. Of course, the case of the VEPTR (Vertical Expandable Prosthetic Titanium Rib) operation should be proven in long-term analyses, as up to now long-term studies are not available [16 -18]. The most recent study (Table 1)on the mid-term results of VEPTR using growing rod instrumentation concerned 19 children with a mean follow-up of 5 years (range: 2 - 7 years). All patients had progressive congenital spinal deformities with failure of segmentation in 5 patients, failure of formation in 4 patients, mixed in 5 patients and unclassified in 5 patients. There were 7 males and 12 females. The major curve Cobb angle improve from a mean of 66 degrees (range: 40 to 95 degrees) preoperatively to a mean of 45 degrees (range: 13 to 79 degrees). The percentage of major curve correction from preoperative to postoperative initial was 31% and from preoperatively to the latest follow-up was 29%. During the treatment period 8 patients (42%) had complications. There were a total of 14 complications (14%). Eleven complications were implant-related (5 rod breakages, 3 proximal construct dislodgements, 2 implant failures and 1 painful protruding anchor that required revision). Three complications included 2 pulmonary complications and 1 postoperative deep infection. These 14 complications required 12 additional procedures. An important finding was the absence of any neurological complications during or after any of the procedure that is of high significance in this higher risk population [70].

To conclude from single case reports that the early fusion prevented the customary severe progression of this condition and early death due to cor pulmonale, somehow seems biased in favour of surgery when, even without surgery, untreated congenital scoliosis would not necessarily lead to cor pulmonale [37,39,40]. It should be acknowledged that the patients reported on in these case reports are not yet over 50 years of age and might develop cor pulmonale in the future.

There is a missing link between the cohorts with only some of the patients beyond the pubertal growth spurt [41-54] and the cases showing possible long-term outcomes [37, 39, and 40]. It would be useful to explore these and other long term effects of surgical treatment in cases of congenital scoliosis in order to allow the therapist a more complete picture of the future development of surgical techniques.

**Figure 4:**
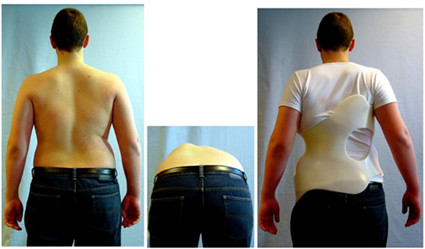
**Congenital scoliosis due to failure of formation with a follow-up of 13 years to Risser 4 under conservative treatment**. Patient with failure of formation and curve 52 degrees at the lumbar spine at the age of 18 months. The patient had not cosmetic complaints. At the age of 18, a small lumbar hump is visible but the patient, finally, does not appears any signs of decompensation.

**Figure 5:**
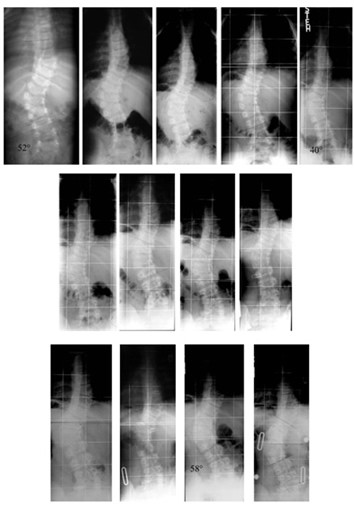
**Congenital scoliosis due to failure of formation with a follow-up of 13 years to Risser 4 under conservative treatment with a brace**. Patient with failure of formation and curve 52 degrees at the lumbar spine at the age of 18 months when brace treatment started. The three rows of x-rays show the complete radiological follow-up: - During the first five years of treatment (first row), the curve has been successfully reduced from 52° (first row left) to 46° (first row middle) at the age of 3.6 years to 40° (first row right) at the age of seven. - Between the age seven to 11 (second row of x-rays) there is no real difference in the follow-up x-rays and the in-brace x-ray (second row on the right) at the age of 11 shows no big correction. - At 13 years progression back to 50° appeared (third row on the left) and the new brace showed only little in-brace correction (third row middle left). The last brace was made at the age of nearly 16 years at Risser 4, when the curve had progressed to 58° after loss of compliance (third row on the middle right). In-brace x-ray showed no real correction in the mature boy (third row on the right).

**Figure 6:**
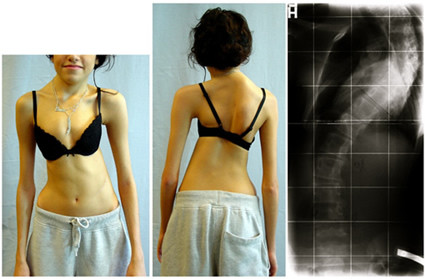
**Patient with failure of segmentation without decompensation due to conservative treatment**. This 18 years old girl presented at the age of 10, with progressive congenital scoliosis with rib synostosis due to failure of segmentation. The patient has denied surgery. During the last follow - up, the clinical appearance demonstrates that a severe decompensation as had to be expected has been prevented. The radiograph demonstrates a curve of 72°. VC was 650 ml, 19% of the predicted value.

With regard to conservative treatment, it should be noted that, here too, few reports exist in the literature. In the case series of three patients with severe congenital scoliosis treated conservatively [14] the patient with a lumbar curve due to hemivertebrae was kept within acceptable functional and clinical limits (Figures 4, 5). On the contrary, of the two other patients suffering from congenital scoliosis due to failure of segmentation in the thoracic region with accompanying rib synostosis, in one severe decompensation has been prevented clinically under conservative treatment, showing no significant trophical limitations of the musculature (Figure 6) although her vital capacity was remarkably reduced. Specifically, follow-up was from the age of 10 to 18 years old. At 10 years, the curve was 62° and progressed to 71° at the age of 12. During the last follow up at the age of 18 years the curve was 72°. The VC (Vital Capacity) was 650 ml, 19% of the predicted value. Severe decompensation was prevented. However, a severe thoracic deformity is evident with underdeveloped lung function and severe restrictive ventilation disorder.

**Figure 7:**
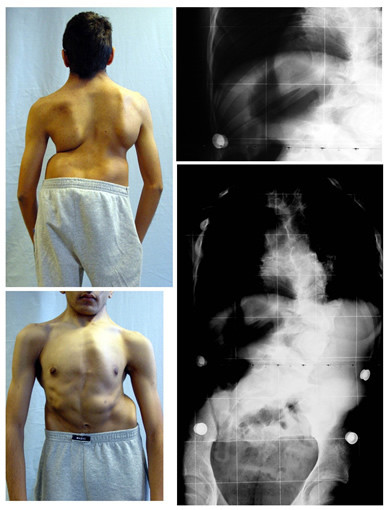
**Patient with failure of segmentation with clinical and radiological improvement due to conservative treatment**. This 15 years old boy presented at the age of 9, with congenital scoliosis with rib synostosis due to failure of segmentation, before entering the pubertal growth spurt. During his last follow - up, his clinical appearance demonstrates a severe deformity. The radiographs demonstrate a scoliotic curve of 59 degrees with Risser sign 4. VC was 1.640 ml, 33% of the predicted value.

The other patient who had refused surgery before pubertal growth spurt, shows clear trophical deficiencies and a very small thorax where cor pulmonale will be easily predictable. Moreover, at the age of 9 years the scoliotic curve was 64°, while at the age of 15 years old with Risser 4 the scoliotic curve was 59°, and no progression has been detected. The ATR (Angle of Trunk Rotation) has been reduced with the Chêneau braces applied from initially 17° to 9° at the age of 16 years, during the last SIR (Scoliosis International Rehabilitation). His VC (Vital Capacity) was 1.640 ml, 3% of the predicted value (Figure 7).

Another two conservatively treated cases of congenital scoliosis caused by wedged vertebrae were reported by Cheneau, Grivas et al [70]. The first patient was an eleven-year-old boy, with a L3 incarcerated hemivertebra, and a 21° Cobb angle between L2 and L4. The second patient was a six year-old girl, with an abnormal block wedge vertebra and a 23° Cobb angle between T5 and T6. They were treated conservatively with a modified Boston brace and a typical Cheneau brace respectively. The long-term follow up revealed that wedged vertebrae were sufficient normalized in both patients and no further treatment was needed [71].

## Conclusions

Due to the limited number of references in the literature, further research is deemed necessary to document whether health-related signs and symptoms improve in the long-term, when spinal fusion is performed in patients with congenital scoliosis. It is also necessary to identify their role in the natural history of congenital scoliosis and their impact on a growing spine.

The same would be desirable for cases where conservative treatment was applied.

Although studies were reported on case series of patients with formation failures which followed conservative approach, its indications are not yet sufficiently documented.

On the contrary, patients with segmentation failures should be treated surgically as early as possible, according to the rate of deformity formation and certainly before pubertal growth spurt to try to avoid cor pulmonale, even though there is lack of evidence for that in the long-term.

## Competing interests

The authors declare that they have no competing interests.

## Authors' contributions

All the authors read and approved the final manuscripts. ΑΚ: Participated in the literature search on PubMed and in drafting the paper. TBG: Participated in the literature search on PubMed and in drafting the paper. HRW: Had the idea for the study, participated in the manuscript drafting, the PubMed search, and provided the figures. DT: Participated in the manuscript drafting, copyediting and research of databases.
